# Nanostructure-induced performance degradation of WO_3_·*n*H_2_O for energy conversion and storage devices

**DOI:** 10.3762/bjnano.9.265

**Published:** 2018-11-12

**Authors:** Zhenyin Hai, Mohammad Karbalaei Akbari, Zihan Wei, Danfeng Cui, Chenyang Xue, Hongyan Xu, Philippe M Heynderickx, Francis Verpoort, Serge Zhuiykov

**Affiliations:** 1 Center for Environmental and Energy Research, Ghent University Global Campus, Yeonsu-gu, Incheon 21985, South Korea; 2 Department of Green Chemistry and Technology, Faculty of Bioscience Engineering, Ghent University, Coupure Links 653, 9000 Ghent, Belgium; 3 Science and Technology on Electronic Test and Measurement Laboratory, North University of China, Taiyuan, Shanxi 030051, P.R. China; 4 School of Materials Science and Engineering, North University of China, Shanxi 030051, P.R. China; 5 National Research Tomsk Polytechnic University, Lenin Avenue 30, Tomsk 634050, Russian Federation; 6 Laboratory of Organometallics, Catalysis and Ordered Materials, State Key Laboratory of Advanced Technology for Materials Synthesis and Processing, Center for Chemical and Material Engineering, Wuhan University of Technology, Wuhan, P.R. China

**Keywords:** 2D layered oxides, interlayer water, van der Waals interaction, WO_3_·*n*H_2_O

## Abstract

Although 2D layered nanomaterials have been intensively investigated towards their application in energy conversion and storage devices, their disadvantages have rarely been explored so far especially compared to their 3D counterparts. Herein, WO_3_·*n*H_2_O (*n* = 0, 1, 2), as the most common and important electrochemical and electrochromic active nanomaterial, is synthesized in 3D and 2D structures through a facile hydrothermal method, and the disadvantages of the corresponding 2D structures are examined. The weakness of 2D WO_3_·*n*H_2_O originates from its layered structure. X-ray diffraction and scanning electron microscopy analyses of as-grown WO_3_·*n*H_2_O samples suggest a structural transition from 2D to 3D upon temperature increase. 2D WO_3_·*n*H_2_O easily generates structural instabilities by 2D intercalation, resulting in a faster performance degradation, due to its weak interlayer van der Waals forces, even though it outranks the 3D network structure in terms of improved electronic properties. The structural transformation of 2D layered WO_3_·*n*H_2_O into 3D nanostructures is observed via ex situ Raman measurements under electrochemical cycling experiments. The proposed degradation mechanism is confirmed by the morphology changes. The work provides strong evidence for and in-depth understanding of the weakness of 2D layered nanomaterials and paves the way for further interlayer reinforcement, especially for 2D layered transition metal oxides.

## Introduction

Within the less than 20 years since the successful exfoliation of atomically thin graphene, 2D layered nanomaterials have been contributing greatly to the advances of nanoscience and nanotechnology with their exotic properties and versatility of applications [1–10]. Among all 2D nanomaterials, 2D transition metal oxides (TMOs) are the group with the highest electrochemical activities for energy conversion and storage [11–15]. As the energy-related field is rapidly developing, the limits of 2D TMOs began to come clear and ways of chemical functionalization were developed to make them more suitable for practical applications [16–18]. However, comparably less attention has been paid to the comprehensive investigation of the disadvantage of 2D TMOs and their failure mechanism.

Tungsten trioxide (WO_3_) is one of the few TMOs with both excellent electrochemical and electrochromic properties [19–23]. It has a three-dimensional (3D) network lattice structure consisting of corner-sharing or edge-sharing WO_6_ octahedra [24–26]. Its phases (monoclinic, triclinic, orthorhombic and tetragonal) form trigonal, quadrangular, pentagonal, and hexagonal tunnels and cavities for 3D electrochemcial intercalation [27–29]. In contrast, its hydrates exhibit a 2D layered strcuture composed of WO_5_(OH_2_) single sheets in a corner-sharing arrangement with additional water molecules between layers, which is suitable for 2D intercalation chemistry [30–32].

Both WO_3_ and its hydrates have been fabricated via different methods and analyzed with regard to electrochemical, photocatalysis, sensing and electrochromic applications [24,33–34]. Oriented WO_3_·H_2_O sheets were hydrothermally grown in mixed acids at 80 °C for 17 h, followed by sintering at 500 °C in order to obtain crystalline WO_3_ for the photoelectrochemical water oxidation [35]. A 2D WO_3_ nanosheet sensor fabricated by high-temperature anodization of tungsten thin films displayed a maximum response of 80% for 1% of hydrogen gas at 250 °C [36]. 2D WO_3_·2H_2_O films developed by a facile dipping process exhibited a significantly improved response time as electrochromic electrodes compared to WO_3_ thin films [37]. The acidic precipitation reaction was also adopted to fabricate WO_3_·2H_2_O electrochemical energy storage electrodes with a higher rate capability than annealed WO_3_ [38]. The investigation of 2D sheets of WO_3_ and a rGO–WO_3_ composite prepared via a one-pot hydrothermal method suggested that the rGO–WO_3_ composite could be a promising material for photocatalytic and antibacterial applications [39]. Unfortunately, despite the great number of 2D WO_3_ compounds and their hydrates synthesized and utilized for energy conversion and storage applications, their weakness has not yet been investigated thoroughly.

In this study, WO_3_·*n*H_2_O (*n* = 0, 1, 2) was fabricated by a facile hydrothermal method for the first time at the different termperatures to investigate the disadvantages of 2D structures. The growth mechanism analyzed by X-ray diffraction (XRD) and scanning electron microscopy (SEM) suggested a 2D to 3D structural transition upon temperature increment, revealing that the weakness of layered 2D WO_3_·*n*H_2_O originates from weak interlayer van der Waals interactions. The faster performance degradation in electrochemical tests of 2D layered WO_3_·*n*H_2_O further indicated the structural instability of 2D nanostructures compared to 3D nanostructures. The structural transformation of 2D layered WO_3_·*n*H_2_O to 3D structures was observed via ex situ Raman measurements under electrochemical cycling experiments. The morphology change confirmed the degradation mechanism proposed in this work. Consequently, this work provides an in-depth understanding of the weakness of 2D layered nanomaterials and paves the way for the interlayer reinforcement of 2D TMOs.

## Experimental

All nanostructured WO_3_ and their hydrates in this work were prepared on FTO/glass substrates through a facile hydrothermal reaction at different temperatures. All chemicals were purchased from chemical suppliers and were used without further purification.

Before the hydrothermal reaction, the seed solution was first spin-coated on the FTO/glass and annealed at 350 °C for 20 min. To prepare the seed solution, 0.824 g of sodium tungstate dihydrate (Na_2_WO_4_·2H_2_O) was initially dissolved into 10 mL deionized water under continuous stirring. After complete dissolution of the Na_2_WO_4_·2H_2_O powder, 0.416 mL of hydrochloric acid (HCl, 36–38 wt %) solution was added dropwise while stirring for 15 min at room temperature. Subsequently, 0.2241 g of oxalic acid (C_2_H_2_O_4_) was added to the solution and the solution was then diluted with deionized water to a total volume of 12.5 mL accompanied by another 15 min of stirring. The prepared seed solution was spin-coated onto FTO/glass at 3000 rpm for four times with each step consisting of 40 s spin-coating at room temperature followed by annealing at 350 °C for 20 min.

The as-prepared FTO/glasses were sealed in 25 mL Teflon-lined stainless autoclaves filled with 15 mL solution that was prepared by the same procedures as the seed solution mentioned above. However, its concentration was ten times lower than that of the the seed solution. Then the autoclaves were heated at temperatures of 80, 100, 120, 150 or 180 °C for 2 h. After heating, the autoclaves cooled down to the room temperature naturally. The FTO/glass substrates were taken out from autoclaves and dried at 60 °C for 1 h. Subsequently, the as-grown WO_3_ and its hydrates were measured and analyzed.

The crystal structures of the samples were identified with a high-resolution X-ray diffractometer (HR-XRD, SmartLab, Rigaku). The morphology of samples was investigated by field-emission scanning electron microscopy (FE-SEM, JSM-7100F, Jeol) together with energy-dispersive spectroscopy (EDS). Information about chemical composition and bonding was collected by X-ray photoelectron spectrometry (XPS, K-Alpha, Thermo Scientific) and Raman spectroscopy (EZRaman-N-785, TSI. Inc.), respectively.

Electrochemical characterization of the samples was performed using an Autolab PGSTAT204 (Metrohm Autolab B.V.) with a three-electrode configuration in 1.0 M H_2_SO_4_ aqueous solution. The as-prepared sample, a Pt wire and a Ag/AgCl electrode acted as working, counter and reference electrode, respectively. Cyclic voltammetry (CV) was conducted in the potential range from −0.8 V to +0.8 V (vs Ag/AgCl).

## Results and Discussion

**Figure 1:**
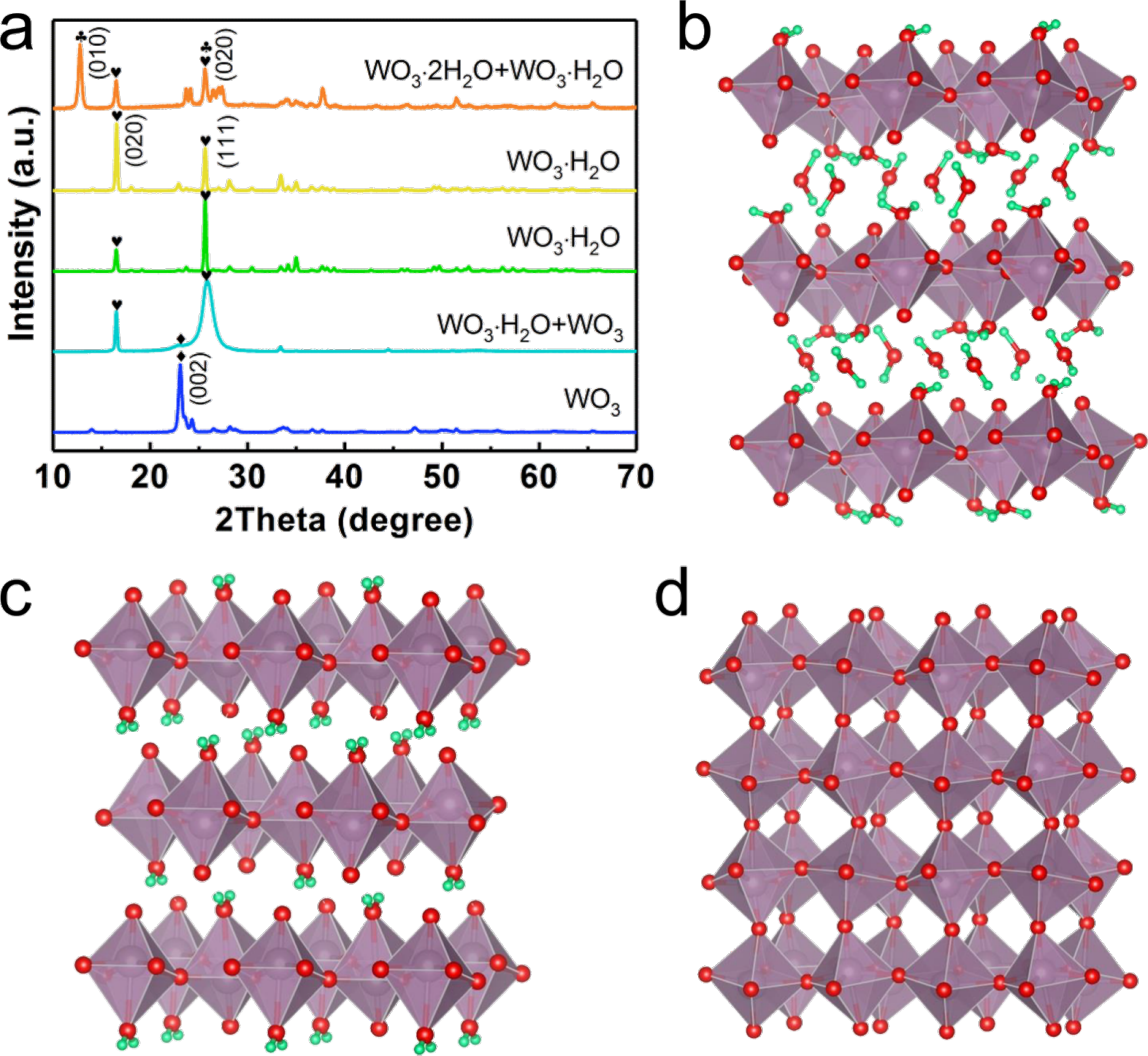
a) XRD patterns of the as-prepared samples at 80, 100, 120, 150 and 180 °C from top to bottom. Schematic illustration of the crystal structures: b) monoclinic WO_3_·2H_2_O, c) orthorhombic WO_3_·H_2_O and d) monoclinic WO_3_.

The crystal structure of the as-prepared samples was initially investigated by XRD. As shown in the XRD patterns from the top to bottom of Figure 1a, the samples display different phase compositions depending on the synthesis temperature. The samples synthesized at 80 °C is composed of monoclinic WO_3_·2H_2_O (JCPDS No. 18-1420) [40] and orthorhombic WO_3_·H_2_O (JCPDS No. 43-0679) [41], which are both layered structures as illustrated in Figure 1b and Figure 1c, respectively. The structural difference between these two components is the number of interlayer water molecules. For the samples synthesized at 100 and 120 °C, only WO_3_·H_2_O (JCPDS no. 43-0679) was measured with two main peaks corresponding to (020) and (111) as some of the interlayer water disappeared [41]. However, the dominant facet changed from (020) to (111) as the synthesis temperature increased. Moreover, for the samples synthesized at 150 °C, the full width at half maximum (FWHM) of the (111) reflection of the main component WO_3_·H_2_O broadened, which indicates the instability of WO_3_·H_2_O at such a high temperature. A small hump on the left side of the (111) peak of WO_3_·H_2_O clearly shows the appearance of monoclinic WO_3_ (JCPDS no. 43-1035) [42–43], which also supports the dehydration tendency of WO_3_·H_2_O. When the samples were grown at 180 °C, only monoclinic WO_3_ (JCPDS no. 43-1035) with its dominant (002) facet existed in the sample. The crystal structure of monoclinic WO_3_ presented in Figure 1d displays 3D covalent bonding structures compared to the stacked 2D layers of WO_3_·2H_2_O and WO_3_·H_2_O with only weak interlayer van der Waals forces. It is also noteworthy that at temperatures lower than 80 °C, neither WO_3_·2H_2_O nor WO_3_·H_2_O could be synthesized using the hydrothermal process presented in this work. Both experimental results and the theoretical models mentioned above suggested that the layered WO_3_ hydrates are relatively unstable compared to WO_3_.

**Figure 2:**
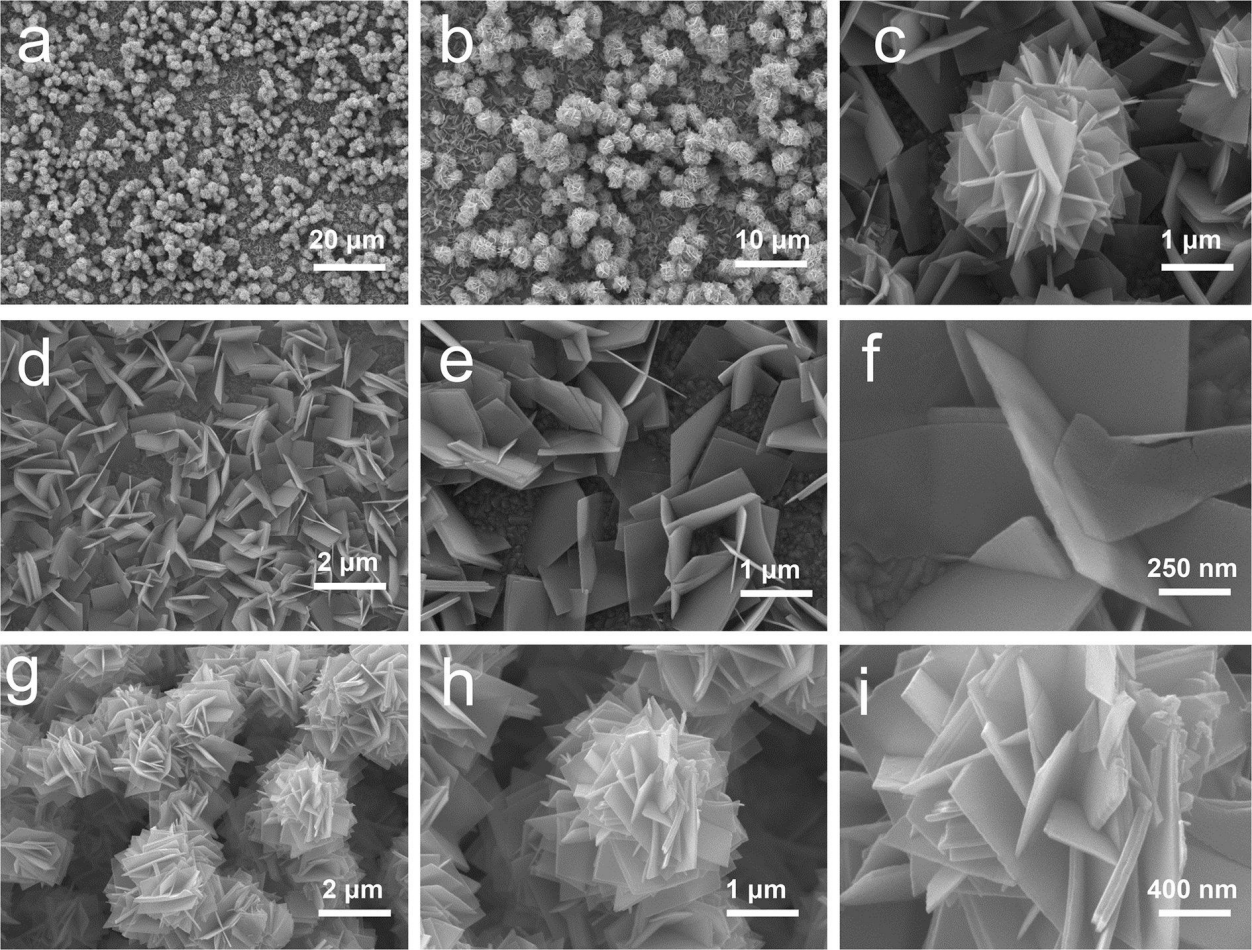
SEM images of the sample synthesized at 80 °C. a) Overview and b) magnified images of the WO_3_ hydrates grown on the substrate; c) image of a representative area; d) typical image of nanosheets and e) magnified view; f) image of a typical nanosheet; g) grouped flower-like balls; h) typical flower-like ball with i) magnified image.

**Figure 3:**
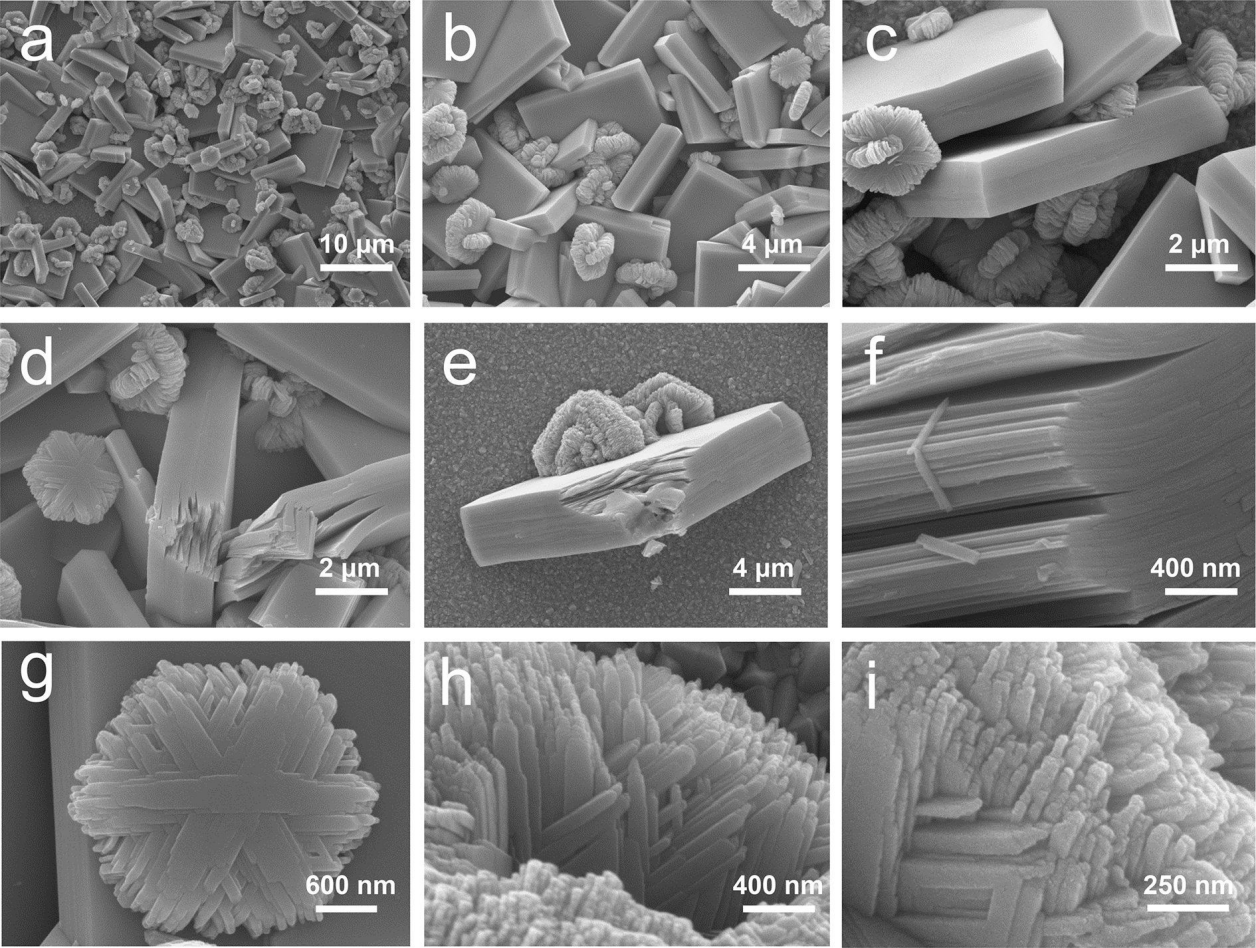
SEM images of the sample synthesized at 120 °C. a) Overview and b) magnified images of WO_3_·H_2_O grown on the substrate; c) image of a representative area; d) square sheets with opened layers; e) a typical square sheet with opened layers and f) its magnified view at the opened layers; g) a typical hexagonal plate; magnified views of h) the edge and i) the side face of a hexagonal plate.

**Figure 4:**
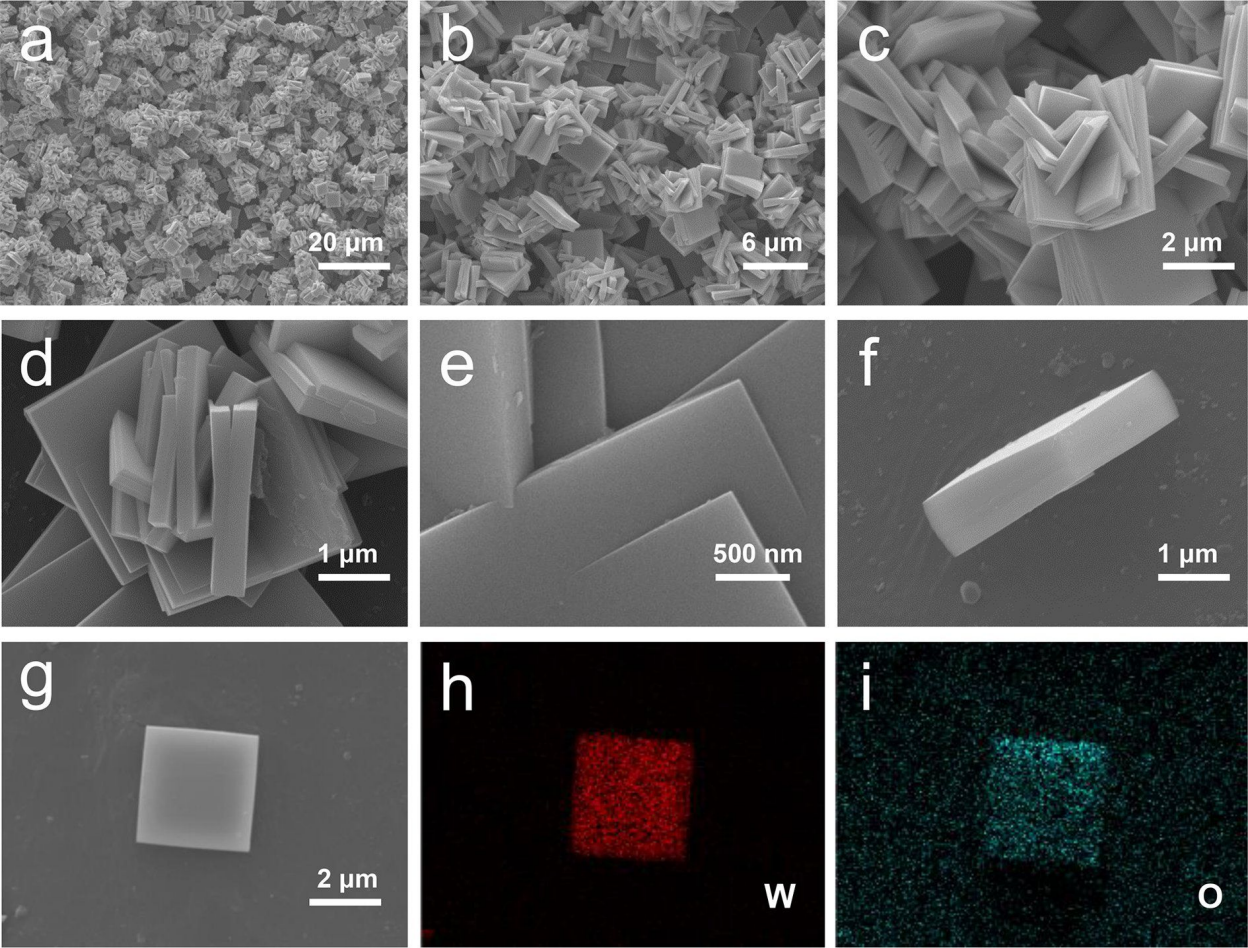
SEM images of the sample synthesized at 180 °C. a) Overview and b) magnified images of WO_3_ grown on the substrate; images of c) one part of the network structure and d) the magnified view; e) image of the crossed nanosheets; f) vertically standing and g) flat laying square sheets. EDS element mapping of the flat laying square sheet: h) W and i) O.

The following figures (Figures 2–4) show the morphology of the three typical samples synthesized at 80, 120 and 180 °C, examined by SEM. Figure 2a,b shows the relatively uniform growth of WO_3_ hydrates with flower-like balls on the upper layer and nanosheets beneath them as represented in Figure 2c. The nanosheets were ca. 1.3 μm square-shaped and almost vertically aligned on the substrate (Figure 2d,e). The image of a typical nanosheet shows the very low thickness of ca. 27 nm (Figure 2f). The upper layer with grouped flower-like balls is highlighted in Figure 2g,h. The images show that the flower-like balls have a diameter of ca. 2.5 μm and are composed of self-assembled square nanosheets with similar sizes as the nanosheets in the layer grown beneath. The magnified image in Figure 2i displays nanosheets with average thickness of ca. 37 nm in a flower-like ball crossing each other.

Figure 3 shows SEM images of the sample synthesized at 120 °C. The as-grown pure WO_3_·H_2_O existed in form of square sheets and hexagonal plates as displayed in Figure 3a–c. The square sheets were measured to be ca. 6 μm long and ca. 1.5 μm thick, while the hexagonal plates were 0.5 μm thick with diagonal length of ca. 2.9 μm. Figure 3d,e shows the square sheets with opened layers, indicating the layered crystal structure of the as-grown material. The magnified image of the opened layers in Figure 3f clearly demonstrated the 2D layered nature of WO_3_·H_2_O. In addition to the square sheets, the hexagonal plates were stacked by nanoribbons in the direction parallel to the diagonals of the hexagons (Figure 3g). As shown in Figure 3g,h, the nanoribbons were wider (ca. 400 nm) at the center and narrower (ca. 100 nm) near the two ends, forming tips at their very ends (Figure 3i).

The SEM images of the sample synthesized at 180 °C are presented in Figure 4. The WO_3_ structures are assembled from square sheets with a few individual sheets beneath as indicated in Figure 4a and Figure 4b. The square sheets with a length of ca. 2.8 μm and a thickness of ca. 0.4 μm grew crossed with each other at all angles, forming a network structure (Figure 4c,d). Figure 4e clearly displays the crossed square nanosheets in the network structure. The individual square sheets were measured to be ca. 3.5 μm long and ca. 0.7 μm thick (Figure 4f,g). The EDS element mapping in Figure 4h,i demonstrates a homogeneous elemental distribution in the sheet and confirms the formation of WO_3_. The oxygen appearing outside the square comes from the substrate.

**Figure 5:**
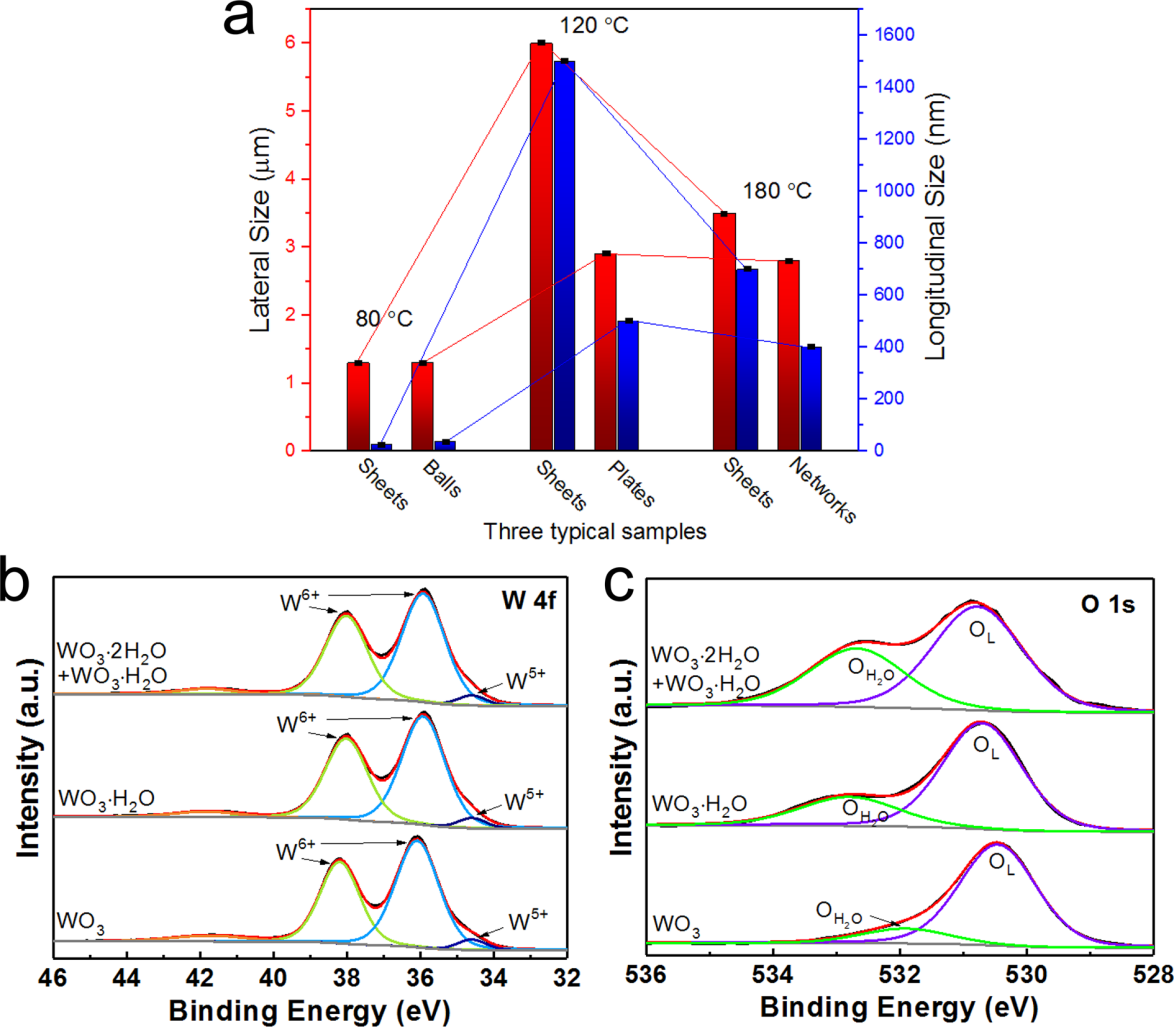
a) The structural feature sizes of three typical samples synthesized at 80, 120 and 180 °C, respectively. High-resolution XPS core-level b) W4f and c) O1s spectra of the three as-synthesized samples.

Three typical sample structures acquired with SEM are presented in Figure 5a. It clearly shows that the feature structure size of the nanostructures synthesized at 80 °C are much smaller compared to similar feature structures obtained in the other two samples, especially in terms of the longitudinal size. Besides the lower synthesis temperature, this could be mainly due to the interlayer water molecules in WO_3_·2H_2_O, which caused the slow growth of the samples. The structural features of the WO_3_ sample synthesized at 180 °C are also smaller than those of the WO_3_·H_2_O sample synthesized at 120 °C. Although a higher synthesis temperature produces more energy for the sample growth, the layered WO_3_·H_2_O still grew faster than the 3D WO_3_, as the formation of van der Waals interactions consumes less energy than the formation of covalent bonds. From both the structural and energy consumption point of view, WO_3_·2H_2_O and WO_3_·H_2_O are kinetically and thermodynamically less stable than WO_3_.

Figure 5b,c depicts the high-resolution XPS core-level W4f and O1s spectra, respectively. The W 4f orbitals in Figure 5b are almost identical in the three samples and can be resolved into W 4f_5/2_ and W 4f_7/2_. The two main peaks correspond to the W4f_7/2_ and W4f_5/2_ of the tungsten atoms in a +6 oxidation state [44–45]. The two peaks from the two samples synthesized with lower temperatures are both located at 35.9 eV and 38.0 eV, which is ca. 0.2 eV lower than the peak locations (ca. 36.1 eV and ca. 38.2 eV) in the WO_3_ sample synthesized at 180 °C. The very small peaks at ca. 34.6 eV in all three samples originate from W^5+^ ions in the lattice, which reveals the formation of a few oxygen vacancies [29,46]. The O 1s spectra for all samples in Figure 5c depict two peaks with one main peak from the lattice oxygen, O_L_, and another from the oxygen in water molecules, O_H2O_ [47–48]. With increasing temperature, the lattice oxygen in the three as-synthesized samples shifts to lower binding energies from 530.8 eV over 530.6 eV to 520.3 eV. The area of the O_H2O_ peaks indicates the structural difference between layered WO_3_·2H_2_O, layered WO_3_·H_2_O and 3D WO_3_. The decreasing area of O_H2O_ peaks with higher synthesis temperature demonstrated the increasing stability of the samples as the interlayer water and coordinated water molecules disappeared successively with only a few unavoidable surface-absorbed water molecules left [49–50]. The above XPS results confirm the SEM analysis.

To get further inside of the electrochemical performance degradation of the three samples, CV tests were carried out at a scan rate of 50 mV·s^−1^ within the potential range from −0.8 V to +0.8 V (vs Ag/AgCl). The electrochemical energy conversion and storage of WO_3_·*n*H_2_O in H_2_SO_4_ electrolyte are based on the intercalation of protons and injection of electrons as described in the following equation [38]:


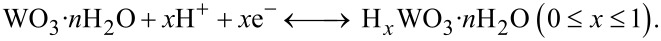


**Figure 6:**
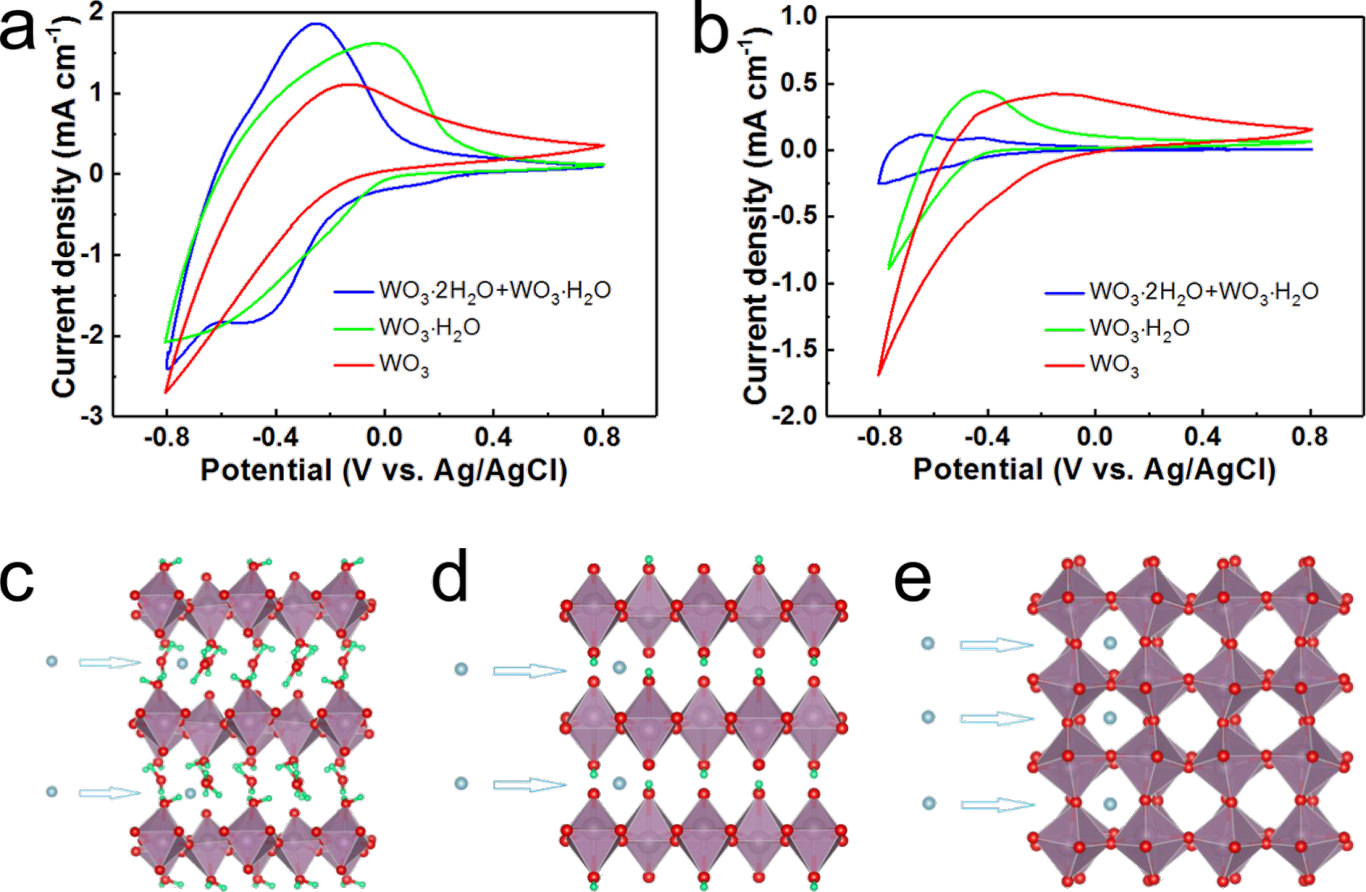
CV curves of the three samples at a) the 1st cycle and b) the 500th cycle; schematic illustration of ion intercalation into c) WO_3_·2H_2_O, d) WO_3_·H_2_O, and e) WO_3_.

The transition of W between the valence states of W^6+^ and W^5+^ is the basis of both electrochemical energy storage and electrochromic behavior. As shown in Figure 6a, in all cases the cathodic current rises when the potential was scanned to negative values because of the intercalation of protons into the samples. Figure 6c–e illustrates the intercalation of ions into WO_3_·*n*H_2_O. The reduction of W^6+^ to W^5+^ in the process also resulted in coloration. In the reverse process, the rise of anodic current indicates the deintercalation of protons and oxidation of tungsten ions with concomitant bleaching. The video presented in Supporting Information File 1 shows the coloration/bleaching processes of the samples during cycling. With a growing number of cycles, the samples exhibited a strong performance degradation as displayed in Figure 6b. The integrated areas of the CV curves in Figure 6b suggested much larger drops of the specific capacitance of the WO_3_ hydrates compared to those of WO_3_. As shown in Figure 6c–e, the intercalated ions in WO_3_ hydrates are surrounded by weak hydrogen bonds, coordination bonds, and van der Waals forces, while the ions in WO_3_ are surrounded by a strong 3D covalent bond network. The intercalation/deintercalation process in WO_3_ may cause less distortions and destructions of the structure than in WO_3_ hydrates, leading to a better electrochemical stability.

**Figure 7:**
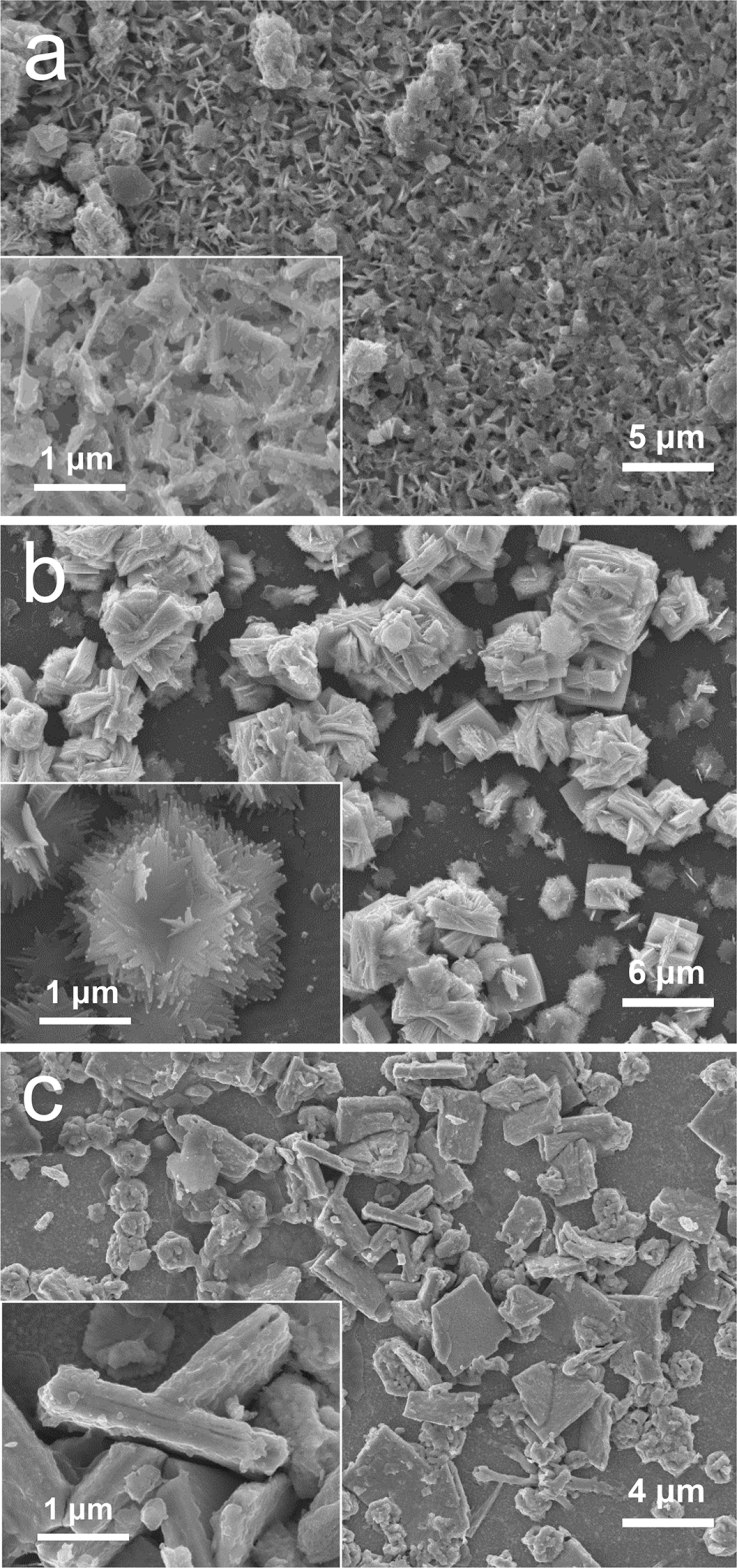
SEM images of the three typical samples synthesized at a) 80 °C, b) 120 °C and c) 180 °C after 500 CV cycles.

The SEM images of the samples taken after 500 cycles of CV support the performance degradation. Specifically, the nanostructured flower-like balls and the nanosheets in the samples synthesized at 80 °C seem to be glued together in Figure 7a, forming a thin film-like structure as indicated in the inset of Figure 7a, which could be a result of strong reaction between the interlayer water molecules and the electrolyte. For the WO_3_·H_2_O sample synthesized at 120 °C (Figure 7b), the sheets were found to swell heavily due to the 2D intercalation/deintercalation processes in the CV cycles, turning almost twice as thick as their original thickness. Under the strong effect of 2D intercalation/deintercalation, the hexagonal plates were transformed into very thin nanosheets, which were composed of nanoribbons (Inset of Figure 7b). In contrast to the two samples mentioned above, the WO_3_ sample synthesized at 180 °C still kept some of the features from the original sample, as depicted in Figure 7c. Although the original network was broken, a considerable number of the sheet components in the samples remained almost unchanged in their original shape (Inset of Figure 7c).

**Figure 8:**
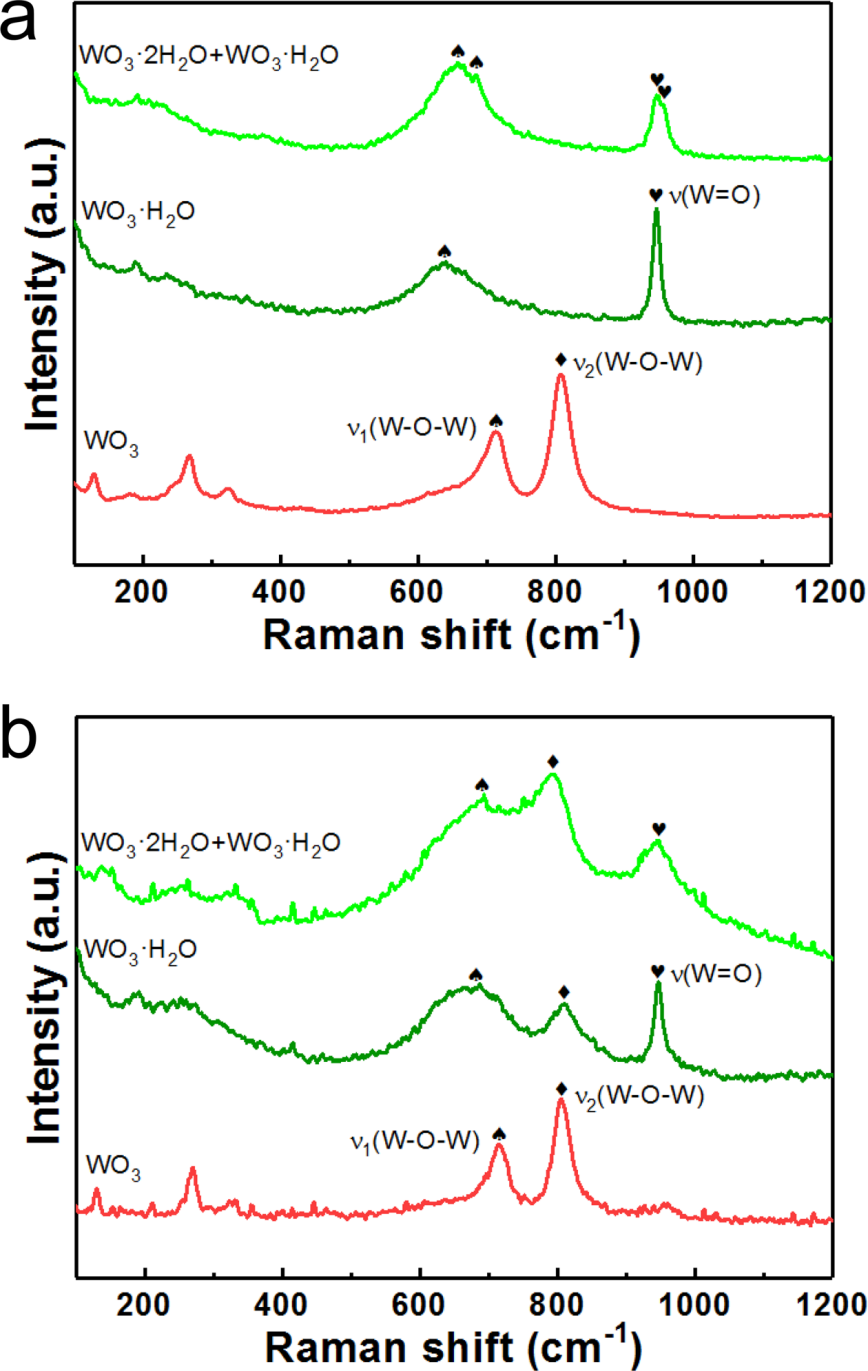
Raman spectra of the three samples a) before and b) after the CV tests.

Figure 8 compares the Raman spectra of the three samples before and after CV tests. As displayed in Figure 8a, the initial WO_3_ sample is characterized by two main peaks at ca. 713 and ca. 807 cm^−1^, which are associated with two types of W–O–W stretching vibration modes [51–52]. The ν_1_(W–O–W) mode also appeared in the other two initial samples. The Raman spectra of the initial WO_3_·H_2_O and WO_3_·2H_2_O samples were characterized by the stretching vibration mode of their terminal W=O bonds [53–54]. In spite of their high similarity, the peaks of WO_3_·2H_2_O are shifted slightly to higher wavenumbers compared to those of WO_3_·H_2_O as indicated in the sample containing both WO_3_·2H_2_O and WO_3_·H_2_O. The Raman spectra presented in Figure 8b reflect the structural transformation of the samples after CV tests. The Raman spectra of both the samples synthesized at 80 and 120 °C showed a new peak from the W–O–W stretching vibration mode (ν_2_(W–O–W)), while the other peaks were broadened. The peaks of the ν_1_(W–O–W) modes in these two samples shifted to higher wavenumbers and became relatively stronger in comparison to the W=O peaks. These results clearly suggest the break of W=O bonds and the formation of W–O–W bonds, which leads to dehydration of WO_3_ hydrates. In contrast, the Raman spectra of the WO_3_ samples synthesized at 180 °C remains almost unaffected after CV tests. The investigation of Raman spectra further supports the relative electrochemical instability of WO_3_·2H_2_O and WO_3_·H_2_O compared to WO_3_.

## Conclusion

In summary, WO_3_·*n*H_2_O (*n* = 0, 1, 2) synthesized in 2D and 3D nanostructures by a facile hydrothermal method, and the disadvantages of the 2D structures were thoroughly examined. The weakness of 2D WO_3_·*n*H_2_O originates from the layered structure. XRD and SEM characterizations of the as-grown WO_3_·*n*H_2_O samples suggested a structural transition from 2D to 3D upon temperature increase. The independent electrochemical tests of three typical samples confirmed a faster performance degradation in the 2D nanostructures compared to 3D nanostructures, supported by the SEM investigation and further explained by subsequent ex situ Raman measurements. Although 2D layered WO_3_·*n*H_2_O nanostructures outranks the 3D network counterparts in terms of the improved electronic properties, they can easily generate the structural instability by 2D intercalation owing to its weak interlayer van der Waals interactions. Their morphology change confirms the degradation mechanism proposed in this work. Consequently, this work provides in-depth understanding the weakness of 2D layered nanomaterials and paves the way for further interlayer reinforcement of 2D TMOs.

## Supporting Information

The video shows the typical coloration/bleaching process of the samples during electrochemical cycling.

File 1 Coloration/bleaching during electrochemical cycling.
